# Sleep disturbances in highly stress reactive mice: Modeling endophenotypes of major depression

**DOI:** 10.1186/1471-2202-12-29

**Published:** 2011-03-24

**Authors:** Thomas Fenzl, Chadi Touma, Christoph PN Romanowski, Jörg Ruschel, Florian Holsboer, Rainer Landgraf, Mayumi Kimura, Alexander Yassouridis

**Affiliations:** 1 Max-Planck-Institute of Psychiatry, Kraepelinstrasse 2, 80804 Munich, Germany; 2 Max-Planck-Institute of Neurobiology, Am Klopferspitz 18, 82152 Martinsried, Germany

## Abstract

**Background:**

Neuronal mechanisms underlying affective disorders such as major depression (MD) are still poorly understood. By selectively breeding mice for high (HR), intermediate (IR), or low (LR) reactivity of the hypothalamic-pituitary-adrenocortical (HPA) axis, we recently established a new genetic animal model of extremes in stress reactivity (SR). Studies characterizing this SR mouse model on the behavioral, endocrine, and neurobiological levels revealed several similarities with key endophenotypes observed in MD patients. HR mice were shown to have changes in rhythmicity and sleep measures such as rapid eye movement sleep (REMS) and non-REM sleep (NREMS) as well as in slow wave activity, indicative of reduced sleep efficacy and increased REMS. In the present study we were interested in how far a detailed spectral analysis of several electroencephalogram (EEG) parameters, including relevant frequency bands, could reveal further alterations of sleep architecture in this animal model. Eight adult males of each of the three breeding lines were equipped with epidural EEG and intramuscular electromyogram (EMG) electrodes. After recovery, EEG and EMG recordings were performed for two days.

**Results:**

Differences in the amount of REMS and wakefulness and in the number of transitions between vigilance states were found in HR mice, when compared with IR and LR animals. Increased frequencies of transitions from NREMS to REMS and from REMS to wakefulness in HR animals were robust across the light-dark cycle. Detailed statistical analyses of spectral EEG parameters showed that especially during NREMS the power of the theta (6-9 Hz), alpha (10-15 Hz) and eta (16-22.75 Hz) bands was significantly different between the three breeding lines. Well defined distributions of significant power differences could be assigned to different times during the light and the dark phase. Especially during NREMS, group differences were robust and could be continuously monitored across the light-dark cycle.

**Conclusions:**

The HR mice, i.e. those animals that have a genetic predisposition to hyper-activating their HPA axis in response to stressors, showed disturbed patterns in sleep architecture, similar to what is known from depressed patients. Significant alterations in several frequency bands of the EEG, which also seem to at least partly mimic clinical observations, suggest the SR mouse lines as a promising animal model for basic research of mechanisms underlying sleep impairments in MD.

## Background

As an adaptation to the regular day/night cycles, organisms synchronize their physical activities, energy metabolism, immune functions and sleep by means of a circadian clock system [1-3]. In addition, individuals continuously face unforeseen short- and long-term changes in the environment called ''stressors'', which can be physical or psychological [4,5]. To adapt to these stressful stimuli, another regulatory system, the stress system, has evolved which senses environmental changes through various sensory organs, processes them in the central nervous system (CNS) and adjusts the CNS and peripheral organ activities to improve chances for survival in nature [6-8]. The major system regulating internal homeostasis is the hypothalamic-pituitary-adrenal (HPA) axis, which accounts for circadian activity and mediates the adaptive response to stressors (see reviews cited above).

The clock and the stress system are both fundamental for survival and thus communicate with one another at multiple levels to adjust numerous physiological activities [1,9-11]. Importantly, dysregulation in either of these systems can lead to similar pathologic conditions, including sleep-related problems and mood disorders such as major depression (MD) [10,12-14].

Therefore, it is not surprising that sleep disturbances are among the most common symptoms of MD and characteristic sleep electroencephalographic (EEG) alterations are one of the most robust predictors of an emergent depressive episode [13,15-17]. Compared to healthy subjects, EEG recordings, which allow to objectively assessing sleep structure alterations, revealed that MD patients often suffer from insomnia and sleep fragmentation. They also show a reduced latency to the first episode of REMS, an increased proportion of REMS (increased REMS density) and reduced slow-wave activity (SWA) during NREMS [12,13,16,18]. Most changes of the sleep EEG pattern prevalent during a depressive episode are moderated or even disappear following successful treatment. Those patients where these sleep EEG abnormalities persist, despite their full clinical remission are at increased risk for relapse into another disease episode [19-21]. Furthermore, healthy high-risk probands with a positive family history of affective disorders also have been found to show an increased REM density, indicating that some of the sleep EEG changes might represent neurobiological vulnerability markers or endophenotypes of the disease [22,23].

Another biological hallmark of MD is a dysregulation of the HPA axis (hyper- or hypo-activity), largely involving pathological alterations in the corticotrophin-releasing hormone (CRH) system [6,13,17,24-27]. Common neuroendocrine symptoms of severely depressed patients include a flattened diurnal rhythm of glucocorticoid (GC) secretion, elevated plasma GC concentrations (hypercortisolism) and adrenal hyperplasia [28-31]. Furthermore, dysfunctional GC receptor-mediated negative feedback regulation of the HPA axis and changes in vasopressin and CRH responsiveness have frequently been described (see reviews cited above). However, it is increasingly acknowledged that the diagnosis of MD encompasses patients, who do not necessarily share the same disease biology, supporting the concept that depression needs to be differentiated accordingly to different causal mechanisms [13,25,26,32,33]. For instance, HPA axis overdrive, related to an enhanced secretion of CRH and an impaired negative feedback via GC receptors, is most consistently observed in patients with psychotic depression. These patients also show the most pronounced sleep-EEG alterations, including disrupted sleep, decreased SWA and short REMS latency. In contrast, patients suffering from the so-called atypical subtype of depression are characterised by markedly reduced activity of the HPA axis, while sleep-EEG data suggest that SWA is not reduced and REMS parameters are not considerably altered in these patients [13,32].

Based on the vital link between stress sensitivity and the development of MD [6,17,24,27,34], a new genetic animal model has recently been established, focusing on alterations in HPA axis reactivity as one of the major endophenotypes in depressed patients [35]. This so-called 'stress reactivity' (SR) mouse model consists of three separate breeding lines selected for either high (HR), intermediate (IR), or low (LR) corticosterone increase in response to a moderate psychological stressor (15-min restraint). Significant differences in the reactivity of the HPA axis between HR, IR (independent "control"-line) and LR mice were already found in the first generation of the selective breeding process and proved to be a highly heritable trait, i.e. the respective phenotype was confirmed across all subsequent generations and could even be increased by assortative breeding [35]. Moreover, results of an extensive behavioral test battery applied to the selected mouse lines as well as neuroendocrine characterisation experiments revealed several phenotypic similarities with changes observed in depressive patients [35-38]. In general, HR animals showed cognitive deficits, particularly in hippocampus-dependent tasks, and were relatively hyperactive in some behavioral paradigms, resembling symptoms of restlessness and agitation often seen in psychotic and melancholic depression [35,36,38]. LR mice, on the other hand, showed clearly more passive coping styles, corresponding to signs of retardation and retreat observed in atypical depression [35]. Furthermore, we found that hyper-responsiveness to stressors was associated with rhythmicity changes (increased physical activity towards the end of the resting period), resembling symptoms like restlessness, sleep continuity disturbances and early awakenings that are commonly seen in psychotic and melancholic MD patients [37]. Additionally, HR mice also showed neuroendocrine abnormalities similar to symptoms of MD patients such as reduced amplitude of the circadian GC rhythm and elevated trough levels [35,37]. Sleep-EEG analyses, furthermore, revealed changes in REMS and NREMS as well as SWA, indicative of reduced sleep efficacy and REMS disinhibition in HR mice [37].

Therefore, in the present study we were interested in how far a detailed spectral analysis of several frequency bands, including a sophisticated event history analysis [39], could reveal further alterations in the sleep architecture of this animal model of affective disorders. This is of particular interest, as such an in-depth analysis of sleep characteristics is often used in human MD patients (see references given above), but to our knowledge has not been applied to characterise sleep in a genetic animal model for extremes in HPA axis reactivity. Thus, our data can contribute to understanding the basic mechanisms underlying sleep disturbances associated with HPA axis dysfunctions and could ultimately be used to elucidate the pathophysiology of sleep impairments in MD patients, including the development of new treatment options for the disorder.

## Results

**Figure 1:**
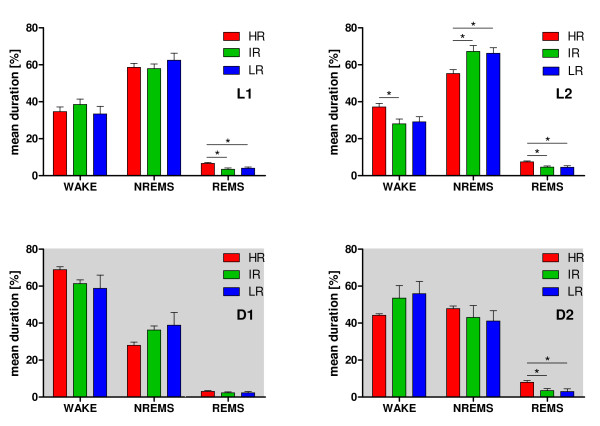
**Mean duration of vigilance states in high reactivity (HR), intermediate reactivity (IR) and low reactivity (LR) mice**. The relative amounts of WAKE, NREMS and REMS are plotted in the panels L1 (light period 1, ZT0-6), L2 (light period 2, ZT6-12), D1 (dark period 1, ZT12-18, dark background) and D2 (dark period 2, ZT18-23, dark background). All three mouse lines display a typical circadian distribution of the vigilance states with significant differences observed in the HR line compared to IR and LR mice. Statistical differences between the mouse lines are indicated by asterisks ((*), Bonferroni modified LSD tests, p < 0.05). Data are given as means ± SEM.

The evaluation of individual vigilance states revealed a typical circadian distribution of the normed durations of WAKE, NREMS and REMS across the recording session in all three breeding lines (Figure 1). During the light periods L1 (ZT0-6) and L2 (ZT6-12), all animals spent more time in NREMS than in WAKE, while especially during the first half of the dark period D1 (ZT12-18) all animals were more awake. During L1, significant group differences were found for REMS among HR, LR and IR animals (F_(2,20) _= 9.535; p = 0.006). Post hoc tests revealed a significantly increased amount of REMS in HR mice, when compared with IR and LR animals (Bonferroni modified LSD-Tests, p < 0.05). In L2, significant group effects were found for all three vigilance states, i.e. WAKE (F_(2,20) _= 6.108; p = 0.03), NREMS (F_(2,20) _= 6.108; p = 0.01) and REMS (F_(2,20) _= 6.108; p = 0.008). Whereas for WAKE the significant group effects were caused only by the differences between HR and LR, for NREMS and REMS the group effects derived from significant differences among all three breeding lines (Bonferroni modified LSD-Tests, p < 0.05). During D1, no differences in mean durations could be found between the groups. During D2, however, strong group differences were found for REMS (F_(2,20) _= 5.573; p = 0.012).

**Figure 2:**
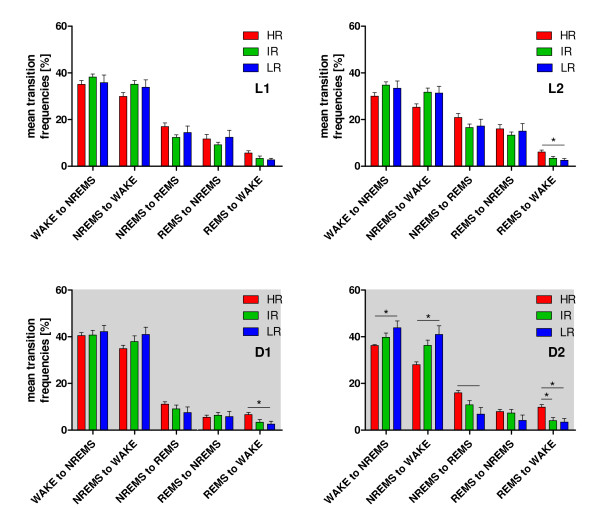
**Mean transition frequencies between the three vigilance states in HR, IR and LR mice**. The relative amounts of transition frequencies for all vigilance states are plotted separately for L1, L2, D1 and D2. In L2 and D1, the HR animals had significantly more transitions from REMS to WAKE, when compared with both other lines. In D2, the HR line showed a different pattern of transitions for all combinations shown, when compared with IR and LR mice, except for the transition of REMS to NREMS. Statistical differences between the mouse lines are indicated by asterisks (*). Data are given as means ± SEM.

**Figure 3:**
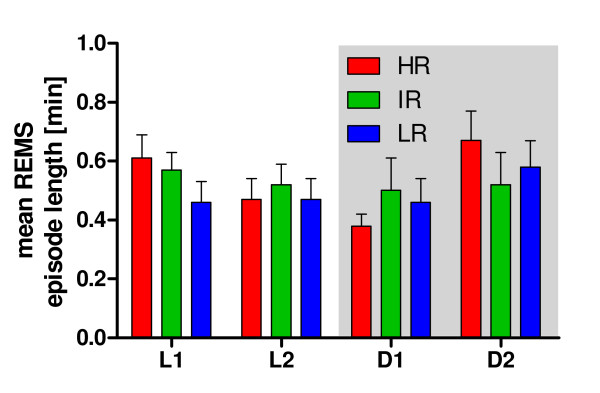
**Mean REMS episode length in HR, IR and LR mice**. Group-comparisons for all three breeding lines during L1, L2, D1 and D2 revealed no significant differences for the mean REMS episode length. Data are given as means ± SEM.

As expected, the most transitions were found between WAKE and NREMS and *vice versa *(Figure 2, L1 - D2). The detailed analysis of the normed transition frequencies calculated for each breeding line revealed significant differences in L2 between REMS and WAKE (F_(2,20) _= 5.163; p = 0.016; HR vs. IR and LR) and in D1 between REMS and WAKE (F_(2,20) _= 4.316; p = 0.028). In general, the fewest transitions were detected between REMS and WAKE (Figure 2, L1 - D2; HR vs. IR and LR). In the time interval D2 group comparisons revealed significant effects on the transition frequencies, which were considerable regarding NREMS to WAKE (F_(2,20) _= 6.632; p = 0.006), REMS to WAKE (F_(2,20) _= 7.322; p = 0.004) and less pronounced regarding WAKE to NREMS (F_(2,20) _= 3.893; p = 0.037) and NREMS to REMS (F_(2,20) _= 5.624; p = 0.012). These transitions showed significant differences preponderantly between HR and LR animals (Bonferroni modified LSD-Tests, p < 0.05). The increased duration of REMS in HR animals, shown in Figure 1 was not a consequence of an overall increased REMS episode length (Figure 3, for L1: F_(2,20) _= 1.339; p = 0.287, for L2: F_(2,20) _= 0.173; p = 0.843, for D1: F_(2,20) _= 0.517; p = 0.605 and for D2: F_(2,20) _= 0.518; p = 0.604) but rather due to an increased number of transitions from NREMS into REMS and back from REMS towards WAKE.

**Figure 4:**
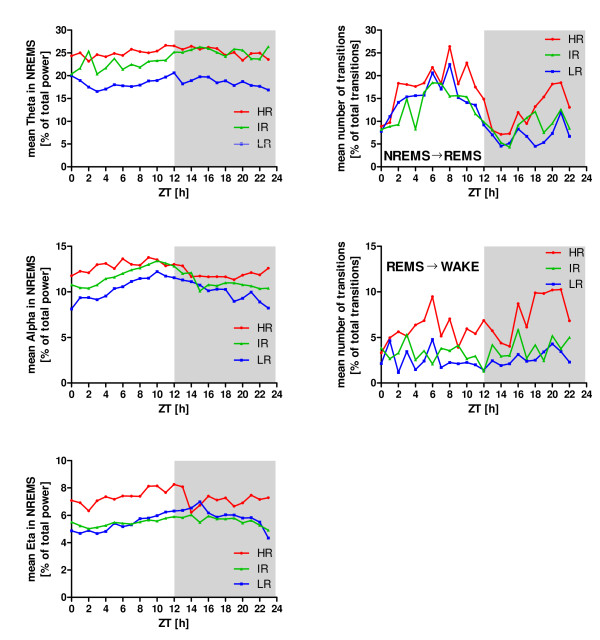
**Stability of progressions over time in HR, IR and LR mice**. Left column: The curve progressions of group means of normalized power (expressed in percent of the total power) are plotted separately for each breeding line during L1, L2, D1 and D2. The mean theta power and mean alpha power of the LR animals clearly showed a stable progression significantly below the mean of both frequency bands in HR and IR mice across the whole experimental time period. Right column: The number of transitions from NREMS to REMS and from REMS to WAKE in HR mice are significantly and robust above the transitions of IR and LR animals. Data are given as means without SEM for reasons of clarity.

After addressing a stability hypothesis on continuity parameters (please refer to the methods section) for particular frequency bands in NREMS (Figure 4, left column), we found that the power of the theta (6-9 Hz) - and alpha-band (10-15 Hz) in LR animals was stable and continuously below the corresponding powers of the other two breeding lines (Wilcoxon matched paired test, p < 0.05). At the eta-band (16-22.75 Hz), a stable differentiation could only be observed between HR and IR animals. For all other frequency bands, no continuously stable differentiations were found, neither in WAKE nor during REMS. After testing the stability on continuity parameters for particular transitions (Figure 4, right column), we found that the frequencies of transitions from NREMS to REMS and from REMS to WAKE in HR animals were stable and continuously above the corresponding transition frequencies of IR and LR (Wilcoxon matched paired test, p < 0.05). All other possible transitions revealed no significantly different continuity parameters.

**Figure 5:**
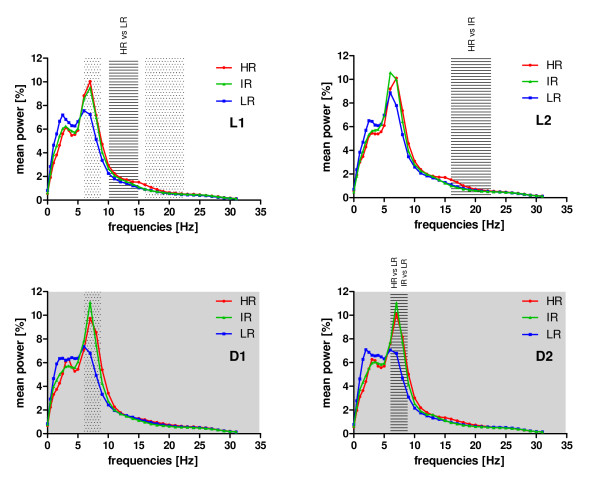
**Mean relative power of the EEG frequency bands in HR, IR and LR mice**. The relative power, summarized for the vigilance states WAKE, NREMS and REMS are plotted separately for L1, L2, D1 and D2. Significant differences in particular frequency bands are highlighted with a striped background (L1: alpha-band at 10-15 Hz; L2: eta-band at 16-22.75 Hz and D2: theta-band at 6-9 Hz). Marginal differences are marked with a dotted background (L1: theta-band at 6-9 Hz and eta-band at 16-22.75 Hz; D1: theta-band at 6-9 Hz). Data are given as means without SEM for reasons of clarity.

Further, we were interested in the distribution of the power of delta (0.75-5 Hz) -, theta-, alpha-, eta- and beta (23-31.75 Hz) -frequency bands in all three breeding lines. Without differentiating between WAKE, NREMS and REMS, during L1 (Figure 5) the mean power of the alpha band revealed a significant group effect (F_(10,32) _= 3.94; p = 0.001), which was attributed to significant differences between HR and LR animals (Bonferroni modified LSD tests, p < 0.05). In L2, the power of the eta band significantly differed between all three groups (F_(10,32) _= 3.99; p = 0.001). Post-hoc Bonferroni tests revealed significant differences solely between HR and IR mice. Whereas in the first six hours of the dark period (D1), no significant differences in any of the considered power spectra appeared, with the power of the theta band within the second half of the dark period (D2) revealing significant distinctions between HR, LR and IR animals (F_(10,32) _= 3.15; p = 0.007). These differences were detected between HR and LR animals and between IR and LR mice (Bonferroni modified LSD tests, p < 0.05).

Additionally, we examined the distribution of the power of delta-, theta-, alpha-, eta- and beta-frequency bands determined in all three breeding lines separately for NREMS and REMS. When considering NREMS during L1, significant effects in several spectra were found between the groups (F_(10,32) _= 3.72; p = 0.002), which were attributed to clear group differences in the theta-, alpha- and eta-band (univariate F-tests in MANOVA). For the power of these bands, the HR and LR animals showed significant differences. Differences were also detected between IR and LR animals, but only for the power of the eta-band (Bonferroni modified LSD tests, p < 0.05). During L2, the power of the spectra in NREMS revealed also significant group differences (F_(10,32) _= 4.22; p = 0.001), caused mostly by the significant differences between HR and LR in the theta-band and between IR and LR in the eta-band (Bonferroni modified LSD tests, p < 0.05). During D1, differences could only be detected for the theta-band (F_(10,32) _= 3.56; p = 0.003) between HR and LR mice (Bonferroni corrected). Finally, during D2 group comparisons revealed also a significant effect on the spectral powers (F_(10.32) _= 4.57; p < 0.001), which was profound regarding the power of the theta and alpha bands (univariate F tests in MANOVA, p < 0.05). The changes in these power spectra were caused by the distinctions between HR and LR for the theta-band and between HR and LR for the alpha-band (Bonferroni modified LSD tests, p < 0.05). When considering all power spectra separately during REMS, we did not find any significant group differences throughout all four time periods L1-D2.

## Discussion

The stress reactivity (SR) mouse model was established as a new mouse model for affective disorders comprising neuroendocrine core symptoms such as a hyperreactive HPA axis, frequently observed in depressed patients [35]. In the present study, we provide evidence supporting the establishment of the SR model as a promising animal model of major depression, especially when the attention is drawn towards disturbed sleep as one of the clinical hallmarks in major depression.

### General sleep/wake behavior

Our experiments showed that all three breeding lines of the SR model, namely the HR, IR and LR line have a typical distribution of sleep and wakefulness across the light/dark cycle with increased amounts of sleep during the light period and of wakefulness and activity during the dark period (Figure 1). This typical sleep/wake pattern was shown for mice in numerous studies. To our knowledge, one of the first studies which investigated this behavior systematically in several nocturnal rodents showed that the sleep time in *Mus musculus *was distributed differentially during the two phases of the light cycle, with more sleep during the light than during the dark phase [40].

The present analysis of the sleep/wake behavior in the SR model (Figure 1) revealed significant differences between the three breeding lines, especially increased amounts of REMS for the HR line during the resting period and the second half of the active period. For HR mice increased GC concentrations during the entire light period as well as at the end of the dark period were shown [37], pointing towards an increased HPA axis activity. It was shown in several studies that CRH, one major player of the HPA axis, can promote REMS. In rats, for example, it was stated that after sleep deprivation with the water-tank method, CRH mediating stress could be the main factor inducing a REMS rebound [41] and suppression of slow wave sleep (SWS) [42,43]. In mice, which conditionally and CNS-restricted over-express CRH, enhanced REMS was attributed to an enhanced CRH secretion [44]. These authors also stated that elevated REMS may serve as a premorbid precursor of hypersecreted CRH predicting a clinical condition such as depression. Similar findings were reported in clinical studies, where the disinhibition of REMS during depression was assigned to a synergism of elevated CRH and cortisol activity [45]. Interestingly, CRH and CRH receptor type 1 and 2 agonists are able to either decrease [46-48] or increase [49] the amount of REMS in both rats and mice. It was concluded that the fragile vigilance state of REMS may be influenced by CRH in opposite directions, depending on the experimental design [46]. Our data from HR mice with a hyperreactive HPA axis also support the notion that elevated REMS is a sign of increased activity of the CRH system.

Increased secretion of CRH in HR animals may also contribute to increased levels of wakefulness in HR animals during the second half of the light period. In rats, restraint stress during the light period resulted in prolonged wakefulness, while CRH receptor antagonists blocked the increase in waking [50]. The reduction of physiologically active CRH levels through application of CRH antisense oligodeoxynucleotides under stress-free conditions led to a reduction in wakefulness, and it was concluded that CRH is involved also in the modulation of spontaneous awaking [51].

The increased amount of REMS in HR mice could not simply be attributed to more REMS *per se*, as a comparison of the mean REMS episode length revealed no significant differences in all four experimental time periods L1-D2 (Figure 3). Rather, the HR line had an increased transition frequency into REMS (from NREMS, although only significant during D2) and from REMS towards wakefulness (Figure 2). These differences in sleep architecture may be partly due to REMS promoting effects of CRH, as discussed above. Accordingly, CRH does not act on duration of REMS episodes but on their amount, leading to an instable and fragmented balance between REMS and wakefulness (Figure 2). Similar sleep alterations were found in a different mouse model of depression. Mice selectively bred for high spontaneous helplessness in the tail suspension test showed enhanced REMS pressure and fragmented sleep [52]. Hyperactivity of the HPA axis, which is observed in depressed patients [53,54], may have been responsible for these physiological changes in sleep/wake behavior, as the helpless mice had elevated serum corticosterone levels [52]. In humans, a causal coherence between impaired sleep and HPA axis hyperactivity was also suggested in the melancholic subtype of depression [45,55].

The increased number of transitions from NREMS to REMS and especially from REMS towards wakefulness in HR animals were consistently above the number of transitions in IR and LR mice across the entire recording time (Figure 4). This points towards a robust phenotype within the sleep architecture of HR animals supporting the applicability of the SR mouse model to mimic sleep impairments in depression such as fragmented sleep.

### Theta enhancement in HR mice

The theta frequency (6-12 Hz, according to different authors) represents the dominant oscillation during REMS [56-59]. It strongly depends on inhibitory GABAergic and excitatory acetylcholinergic inputs from the medial septum into the hippocampus [56,60]. It has been shown that CRH can modulate cholinergically driven hippocampal theta frequency [61] or even enhance theta amplitude and frequency [62]. Interestingly, although the cholinergic and the CRH system seem to interact, CRH enhanced pharmacologically induced cholinergic blockades, leading to impairments during spatial learning [63]. Increased amounts of CRH may, on the one hand, be responsible for enhanced theta activity in HR animals, compared to LR mice (Figure 5), probably causing deficits of HR animals in object memory and spatial memory tasks [38]. Partly not in accordance with this are findings that, although IR animals had increased theta activity, compared to LR mice but not to HR animals (Figure 5), this breeding line did show only intermediate levels of corticosterone when all three lines (HR vs. IR vs. LR) were compared [35] and spatial memory performance in IR was not as affected as in HR mice [38]. Although it is well established that patients with major depression show cognitive impairments [64,65], such symptoms often outlast a recovery from psychopathological symptoms and it seems unlikely that solely hypercortisolism causes these disabilities [66]. It will need to be clarified, in how far cognitive impairments are due to hypercortisolemia-induced long-term changes in relevant brain regions such as the hippocampus [66]. In general, the above outlined interpretation of our data must be critically evaluated in future experiments, since theta enhancement in HR mice may have also been partly caused simply by increased amounts of wakefulness (Figure 1, L2) and REMS (Figure 1, L1, L2 and D2).

### Differentiated alpha frequencies

Cortico-cortical and thalamo-cortical circuits were thought to provide neuronal origins of oscillatory EEG alpha activity [67,68]. More recent publications attribute the alpha band to interconnected populations of layer V neurons from the occipital cortex [69].Modulation of these generators may be realized at the thalamic level, induced by the reticular activating system within the brainstem [70]. The locus coeruleus (LC), as part of the reticular activating system projects to the thalamus (additionally to the forebrain, cortex and hypothalamus) and activation through CRH leads to an increase in EEG frequency, decreased SWS (0.5-4.5 Hz) and increased wakefulness [71,72].Interestingly, LC neurons also discharge maximally during stressful conditions through local release of CRH [73-75].

Increased alpha bands were not only present in HR animals during the first half of the light period, but also in humans in conjunction with depression [76]. These authors classified participants in their study in a high vs. low depression group, according to their scores on two different inventories and found that high depression participants had more alpha activity (8-13 Hz) in frontal and parietal regions of the EEG. As predicted for depression, when differentiating between the two hemispheres of the cortex, less right frontal alpha activity was associated with better memory performance in the low depression group [76] (for the relationship between hemispheric EEG-asymmetry and different aspects of emotion, refer to e.g. [77]). Increased alpha activity in HR animals, when compared with LR animals may also contribute to memory deficits found in HR mice, thus supporting the notion that the SR model is a promising animal model for cognitive deficits in depression [36,38].

### Increased eta power in HR mice

Moreover, we found that HR animals had increased eta power (16-22.75 Hz) during the second half of the light period. The eta band as investigated in our study represents a section of the frequency range normally described as beta band in literature (beta can range from 12 Hz to 35 Hz, depending on individual settings of the experimenter and the species under investigation). Historically, the beta band was set at 12-30 Hz [78]. We introduced the eta band in earlier studies to specify the delta and the beta band during data analysis according to the species-specific EEG of mice (for a precise description of the algorithm, refer to [79]), as opposed to the suggested specifications for rats [80]. The narrow specification of our eta band is hardly found in the literature, when oscillations are discussed; thus, every conclusion from our data on increased eta power would be speculative. Interestingly enough, in humans faster frequencies (the authors defined beta as 14.75-30 Hz) were stated to be indicative of cortical arousal [81], supporting the CNS hyperarousal hypothesis for insomnia [82,83], and beta-2 oscillations (20-30 Hz, as stated by the authors), recorded from the somatosensory and motor cortices were coherent with muscle electrical activity [81]. Our data on increased eta activity (as part of beta) during L2 in HR mice (Figure 5) may be linked to the increased wakefulness detected during L2 (Figure 1).

### Stability of data across recordings

The robustness of the above described theta, alpha and eta differences were predominantly found during NREMS with high stability across the whole recording time (Figure 4), whereas no robust differences for these frequency bands could be observed during REMS (data not shown). It is tempting to speculate that HR mice with robustly increased alpha and eta power, although not dramatically more awake than IR and LR mice during L1-D2 have a shallower NREMS. This is in accordance with clinical studies describing shallow sleep in depressed patients [45,84]. The stability in the progression of increased theta power in HR mice across the whole recording period (Figure 4) could, at least partly, explain the increased amounts of REMS during L1, L2 and D2, also mirrored in the increased transition frequencies from REMS to wakefulness in L2, D1 and D2. In contrary to increased theta, over time robustly reduced amounts of this frequency band, as detected in LR animals may serve as a driving force to decrease REMS.

In general, the beta and delta bands are very much parallel among humans and primates, but one has to be very critical in transferring findings from a rodent EEG to a human EEG, especially for the alpha and theta bands.

## Conclusions

HR mice, i.e. those animals that have a genetic predisposition for hyper-activating their HPA axis in response to restraint stress, showed altered sleep architecture, similar to what is known from depressed patients. Moreover, robust differences along the activities of several frequency bands of the EEG, which also seem to at least partly mimic clinical observations, suggest the stress-reactivity mouse model understanding the mechanisms underlying sleep impairments in patients suffering from stress-related diseases. The herein reported findings also underscore the notion that sleep-EEG measures in rodents could help to find human biomarkers in the future which might provide tools to create patient subgroups with similar, if not identical disease mechanisms.

## Methods

### Animals and housing conditions

All animals used in this study derived from the seventh generation (Gen VII) of the 'stress reactivity' (SR) mouse model [35]. This model was generated from the CD-1 mouse strain and consists of three independent mouse lines selectively bred for either high (HR), intermediate (IR) or low (LR) reactivity of the HPA axis (for a detailed description see [35]. Eight adult males of each breeding line (HR, LR and IR) were used in the experiment. Each animal was housed separately in an individual recording cage located in a sound attenuated chamber at constant laboratory conditions (23 ± 1°C, 12-12 h light-dark cycles, lights on: 10 am). Food and water were available *ad libitum*.

### Surgery

An isofluran/oxygen anesthesia (custom made vaporizing device) was used during all surgical preparations of the animals. Prior to the surgery, each animal received atropine sulfate (0.05 mg/kg BW) and meloxicam (0.5 mg/kg BW) subcutaneously. Each animal was surgically equipped with four epidural electroencephalogram (EEG) and two intramuscular electromyogram (EMG) electrodes. A detailed description of the surgeral procedure is described elsewhere [37,79]. Briefly, to place the EEG electrodes on the cortex, four small holes were drilled into the skull preserving the cerebral membrane. Two electrodes were bilaterally placed at the standard frontal region of the skull (caudal third of os frontalis), one reference-electrode was placed at the right standard parietal area and the ground electrode was inserted at the left standard parietal area (center of os parietalis). Both frontal electrodes where referenced to the right parietal electrode. The contrallateral lead was analyzed, the ipsilateral lead only served as additional recording track, if the standard lead could not be analyzed. Two EMG electrodes were embedded bilaterally of the spine into the neck muscles. Postoperative the animals received meloxicam added to the drinking water (0.5 mg/kg BW). After surgery, the animals were allowed to recover for two weeks in individual recording cages with recording cables attached to them before two successive 23 hour recording sessions of EEG and EMG signals were performed. Each recording cable was connected to a swivel system allowing free movements of the animal.

### Data processing

EEG and EMG signals were fed online into a differential preamplifier (1000x, custom made) and main amplifier (10x, custom made). The EEG signals were analog band-pass filtered (0.5 - 29 Hz, filter frequency roll off 48 dB/octave) and digitized with a sampling rate of 64 Hz (AD board, NI PCI-6070, National Instruments, Austin, USA). Root mean square was applied to all non-filtered EMG signals before its digital conversion (sample rate: 64 Hz). Fast Fourier transform (FFT) for the spectral analysis was applied on consecutive 4 sec intervals. The vigilance states WAKE, NREMS and REMS in each of these intervals were pre-scored semi-automatically with a LabVIEW-based sleep scoring program (SEA, Koeln, Germany) and were rescored manually. The frequency bands applied to the semi-automatic scoring algorhythm and for statistical quantifications were as follows: delta: 0.75-5 Hz; theta: 6.0-9.0 Hz; alpha: 10.0-15.0 Hz; eta: 16.0-22.75 Hz; beta: 23.0-31.75 Hz. The applied scoring algorhythm was originally based on experiments with rats [80]; the eta band was additionally specified in our experiments [79] to meet the criteria for the slightly different mouse EEG. A detailed description of the scoring procedure is described elsewhere [79].

### Statistical analysis

In general, statistical evaluations and the corresponding graphs refer to four separate time intervals of the total recording time: L1 (first half of the light period: ZT0-6 [h]); L2 (second half of the light period: ZT6-12 [h]); D1 (first half of the dark period: ZT12-18 [h]) and D2 (second half of the dark period: ZT18-23 [h]). Of primary interest was the investigation of group effects on the relative stay in the three different vigilance states (WAKE, NREMS, REMS) as well as on the relative transition frequencies between the vigilance states within each of the mentioned time intervals (Note: the relative stays in WAKE, NREM or REMS refers to the total stay, which is equal to the length of the considered time interval and the relative transitions refers to the total transitions within the corresponding time intervals. Such relative quantities are often called normed quantities). Group effects on the normed durations of individual vigilance states and normed transition frequencies were statistically analysed by multivariate analyses of variance (MANOVAs) for each time interval separately. In cases of significant main (global) group effects, univariate F tests followed to identify those variables on which the group effect was significant. For these variables post-hoc tests (Bonferroni modified LSD tests) were subsequently applied for investigating the significance of contrasts or simple effects i.e. of the differences between group pairs in the variables showing main group effects. Group comparisons of the mean REMS episode length were performed individually for L1-D2 by means of one-way analyses of variance. For evaluating group differences in the power of the known frequency bands (delta, theta, alpha, eta, beta) determining in each of the vigilance states the area under the curve (AUC) in each time interval using the trapezoid rule was first calculated and then analyses of variance were performed.

Finally the assumption that for particular groups the frequency bands across the experimental time lie constantly over those of other groups (known as stability hypothesis [85]) was tested for significance with a Wilcoxon Matched-Pairs Signed-Ranks test by considering the part of each mean power above the common median at each time point (ZT1-ZT23). The nominal level of significance was accepted with p < 0.05. To keep the type I error less or equal to 0.05, all posteriori tests (univariate F tests, tests with contrasts) were performed with reduced levels of significance (adjusted α according to the Bonferroni procedure). All data are expressed as mean ± SEM.

### Animal care

Laboratory animal care and all experimental procedures were conducted in accordance with the regulations of the current German Animal Protection Act. The protocols were approved by the Government of Bavaria (AZ209.1/211-33/04 and AZ55.2/1/54-2531-64/07).

## Authors' contributions

Conceived and designed the study: TF CT AY. Performed the experiments: FT CT JR CPNR. Analyzed the data: TF CT AY. Reviewed the manuscript: HF RL CPNR MK. Wrote the paper: TF CT AY. All authors read and approved the final manuscript.
